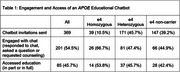# Patient utilization of an educational chatbot to support *APOE* genotyping in the clinical setting

**DOI:** 10.1002/alz70858_107211

**Published:** 2025-12-26

**Authors:** Elisabeth Wood, Meron Azage, Laynie Dratch, Rajia Mim, Demetrios Ofidis, Phillip Trieu, Aysha Syeda, Ethan Broaddus, Sarah Milinski, Brian L Egleston, Sanjeev Vaishnavi, Angela R. Bradbury

**Affiliations:** ^1^ University of Pennsylvania, Philadelphia, PA, USA; ^2^ Fox Chase Cancer Center, Philadelphia, PA, USA; ^3^ Perelman School, University of Pennsylvania, Philadelphia, PA, USA

## Abstract

**Background:**

*APOE* genotyping is recommended for patients considering anti‐amyloid antibody therapy (AAT). It is also recommended that genetic testing for Alzheimer's disease (AD) should be conducted with the support of genetic counseling, as genetic information can raise questions and has implications beyond the initial clinical indication. The advent of AAT has caused an increased demand for both genetic testing and counseling for AD clinical care. Digital tools are a possible solution to meet this demand.

**Method:**

Utilizing prior research and clinical experience, a custom chatbot was designed to provide educational information on AD and the *APOE* gene as a quality improvement project. The chatbot also provided the option to request assistance from a genetic counselor. Electronic health records were queried for patients who received *APOE* genotype results as part of a work‐up for potential treatment with AAT. The chatbot was deployed to the patient's mobile phone number of record. Descriptive statistics were used to analyze utilization of the chatbot. Exit interviews were conducted by telephone with a subset of patients as part of rapid‐cycle‐testing.

**Result:**

The *APOE* educational chatbot was launched to 369 patients, with 201 (54.5%) engaging with the chat (Table 1). Of those who engaged, 85 (45.7%) accessed the educational content in part or full, and 7% sent individual questions through the chat and/or requested genetic counseling given remaining informational needs. Engagement and access varied by genotype: e4 homozygotes had 66.7% engagement, 53.8% access, e4 heterozygotes had 47.4% engagement, 45.7% access, and non‐carriers had 44.9% engagement, 42.4% access. Exit interviews (*n* = 22) revealed high patient value and constructive suggestions for improving the chatbot.

**Conclusion:**

Engagement with a chatbot for *APOE* education was consistently high across all *APOE* result types, suggesting that patients receiving *APOE* genetic testing for the consideration of AAT have interest in *APOE* information regardless of genotype. Patient feedback was positive and constructive, supporting patient value and providing direction for changes to improve utilization. AD clinic populations may find chatbots to be an acceptable method for receiving genetic education about *APOE* and requesting additional support from a genetic counselor when needed.